# Supplementary material for: Are carbon emissions trading and green financial instruments synergistic? -Comprehensive quantitative research based on content analysis
Source: PLoS One. 2024 Mar 7;19(3):e0298601. doi: 10.1371/journal.pone.0298601 (PMC10919601; doi:10.1371/journal.pone.0298601)
Supplement: S1 Data — (ZIP) [file pone.0298601.s001.zip › Data/地级市Z_F工作报告.zip!/╦─┤¿/╡┬╤⌠╩╨/└┤╫╘╣1⁄2╓┌║┼í╛┬φ┐╦╩2╛▌═°í┐_2015.docx]

各位代表：

我代表市人民政府，向大会报告工作，请予审查。

2014年工作回顾

过去的一年，是全面深化改革的开局之年。面对复杂严峻的宏观经济形势和繁重艰巨的改革发展任务，在省委、省政府和市委的坚强领导下，在市人大、市政协的监督支持下，全市上下认真贯彻党的十八大和十八届三中、四中全会精神，深入实施全省“三大发展战略”和全市“一三五八”发展战略，统筹推进“稳增长、调结构、促改革、惠民生”，迎难而上，奋发有为，全市经济社会保持良好发展势头。

一、保持专注发展定力，经济平稳较快增长

积极应对宏观经济下行压力，出台稳增长措施31条，经济发展实现稳中有进。全市实现地区生产总值1515.6亿元，增长9%；一般公共财政预算收入83.5亿元、增长3.8%。

产业发展势头较好。工业经济较快增长，规模以上工业增加值增长10.8%、增速提升6位，新增规模以上工业企业99户、总数达1227户。广汉、什邡、绵竹、中江被确定为全省首批工业强县示范县（市）。服务业有新提升，省级现代服务业综合改革试点工作扎实推进，成功举办第七届中国国际中小企业交易会，服务业增加值增长8.8%。农业稳步发展，全年粮食总产量188.4万吨、增加3760吨，预计肉类总产量38.8万吨，新增农民合作组织187个。

投资拉动明显增强。完成全社会固定资产投资894.9亿元、增长10.8%，142个省市重点项目全部完成年度投资目标。完成工业投资441.4亿元、增长9%，天成不锈钢、信义玻璃等重大项目竣工投产。完成服务业投资438.1亿元，增长14.4%。金融支持实体经济力度加大，中小微企业贷款余额269.05亿元，比年初增加44.7亿元。成功引进长城资产管理公司与德阳银行开展战略合作。金融机构本外币各项存款余额1929.3亿元，比年初增长7.2%；各项贷款余额1106.1亿元，比年初增长10.6%。

消费市场保持活跃。实现社会消费品零售总额529亿元，增长13.1%。参加“川货全国行”、“惠民购物全川行”活动取得显著成效。文化休闲、网络购物等新型消费升温。举办“迎春购物月”、“美食文化节”等节假日促销活动，带动消费15.3亿元。旅游业快速发展，新增国家级4A级景区1个，举办全市第四届旅发大会，实现旅游总收入120.6亿元、增长30.2%。

二、加大结构调整力度，转型发展步伐加快

狠抓产业升级、城市转型、环保攻坚，质量型增长、内涵式发展迈出积极步伐。

结构调整扎实推进。三次产业比重为13.4:60.2:26.4，二、三产业比重分别提高0.1和0.4个百分点。老工业基地调整改造有序推进，关闭和淘汰落后产能企业36户。机械、食品、化工等传统产业改造力度加大，完成技改投资329.3亿元，增长17.3%。战略性新兴产业发展向好，航空与燃机、新能源汽车、页岩气和节能环保产业跻身全省五大高端成长型产业，“东汽重型燃气轮机自主研发项目”与“明日宇航机匣中心和数控中心项目”被列入省56个重大产业项目。现代物流、电子商务等新兴先导型服务业发展加快，4个项目被纳入省50个服务业重大项目。西部特色农产品深加工基地建设成效明显，规模以上农产品加工企业销售收入592.47亿元、增长17.68%。

创新驱动战略深入实施。新增28家高新技术企业，预计实现高新技术产业总产值890亿元、增长20.8%、总量居全省第三。新增国家级科技企业孵化器1个、省级企业技术中心5户，获省科技进步奖17项。新增中国驰名商标5件、四川名牌产品21个。政产学研合作不断深入，与中科院成都分院共建德阳中科先进制造创新育成中心，成立了新能源汽车产业创新联盟。科技金融进一步融合，8家科技型企业在区域性股权交易市场挂牌。推进科技成果转化，新增专利实施项目331项，实施国、省科技计划项目109项，技术合同交易额增长98.8%。“双百工程”成效明显，前两批双百人才研发项目达38个。广汉成功创建“国家知识产权试点市”。

城乡建设加快推进。全市城镇化率达47.27%，提高1.41个百分点，新增建成区面积4.4平方公里，其中市区新增2.38平方公里。交通基础设施更加完善，成南铁路、成德大道、绵茂公路、中金快速通道等项目加快推进，旌江快速干线和广青公路三期完工通车，建成通乡通村公路254公里。华强沟和凯江湖水库开工，64个重点水利项目完成投资12.83亿元。中心城市和城市新区建设加快，79个市政基础设施项目完成投资28.9亿元，东一环路即将全线贯通。10个“百镇建设试点镇”完成投资6.4亿元，开工“美丽乡村”示范村庄47个、竣工15个。实施户籍等“五项改革”，新型城镇化体制机制进一步完善。

生态环保不断进步。成功申报国家资源综合利用“双百工程”示范基地，入选国家节能减排财政政策综合示范城市。成功创建国家森林城市，国家卫生城市通过专家评审。万元工业增加值综合能耗下降率提前完成“十二五”目标，预计单位GDP能耗下降2.5%，主要污染物总量减排可全面完成。大力开展大气污染综合治理，实施大气污染应急攻坚，秸秆禁烧和综合利用成效显著。狠抓沱江流域水污染整治，完成重点治理项目26个。绿化造林稳步推进，森林覆盖率提高0.5个百分点。治理水土流失面积125.6平方公里，新增耕地1.15万亩。

三、全面深化改革开放，发展活力不断释放

精心设计改革方案，以点带面、重点突破、整体联动，有效推进改革开放各项工作。

全面深化改革扎实开局。出台13个专项改革实施方案，公立医院、职业教育等6项改革被列为国家试点。简政放权力度加大，调整和取消222项行政审批项目，停收和免收22项行政事业性收费。加快机构改革，完成市卫计委、市工商局等机构调整和职能整合。国资国企改革稳步推进。深化财税体制改革，国有资本经营预算实现全覆盖，“营改增”试点顺利推进。工商登记制度改革全面启动，新登记公司和注册资本分别增长58.6％、118.2％。加快公共资源交易改革，推行工程招投标、政府集中采购电子化交易。农村改革取得新进展，农村土地承包经营权确权颁证完成90%工作量，农村房屋所有权确权颁证试点工作全面完成，罗江农村商业银行正式挂牌。

开放水平日益提高。招商引资到位内资680.1亿元、增长0.7%，到位外资2.1亿美元；完成进出口总额38.8亿美元、增长14.5%，其中出口31亿美元、增长31.8%、保持全省第二位；对外投资1730万美元。开放载体逐步完善，综合保税区项目纳入国家发改委西部大开发重点项目投资指导目录，推出了全省首个小微企业短期出口信用保险政府统保平台，德阳海关、出入境检验检疫局开关开检。外事、侨务、对台工作取得新进展。

区域合作不断深化。成德同城化步伐加快，编制完成《成德同城化空间发展规划》，成德绵乐城际铁路通车，德阳银行、交通银行、华夏银行、渤海银行实现成德同城化结算。成德工业园投产规模以上工业企业达25户。广汉与青白江、新都打造边界合作实验区，什邡、绵竹与彭州等地环龙门山旅游合作不断深化。对口支援若尔盖县、阿坝县工作深入推进，42个援建项目完工18个，德阿产业园落户绵竹。对口支援芦山县灾后重建扎实推进，援建的芦山产业集中区标准厂房投入使用。

四、推动民生持续改善，社会事业全面发展

千方百计保障和改善民生，城镇居民人均可支配收入26998元，增长9.3%；农民人均纯收入11260元，增长11.5%。

民生工程深入实施。“十项民生工程”共投入资金70.05亿元、比计划增加4.54亿元，新增17件民生实事。全市城镇新增就业3.68万人，城镇登记失业率3.6%。2011级藏区“9+3”免费教育计划毕业生初次就业率达95%。资助普通高中、中职学校家庭经济困难学生16961人和5053人。积极化解“大班额”问题，开工义务教育学校教学用房12863平方米。城乡医疗救助24.88万人次，发放医疗救助金8566.74万元。县级儿童福利指导中心实现全覆盖。大力实施“百万安居工程”，开工保障性安居工程19789套、竣工9528套，为农民工配租公共租赁住房1354套。扶持5.02万农村贫困人口改善生产生活条件，解决10万农村人口饮水安全，完成1859户农村危房改造。

社保水平稳步提升。整合城乡居民基本养老保险制度，新增城镇职工基本养老保险参保3.06万人，城乡居民基本养老保险参保人数达142.2万人，办理城乡居保转企业职工基本养老保险4500人。医保水平进一步提高，新增城镇基本医疗保险7.72万人，初步实现参保人员省市内异地就医即时结算，城镇居民和新农合基本医疗筹资标准提高到390元/人，城乡居民大病保险赔付比例达71.7%。推进社会养老服务体系建设，新增（改造）养老床位3750张。提高了城市低保、农村低保、企业退休人员养老金和最低工资标准。

公共服务均衡发展。新（改）建幼儿园6所。绵竹、广汉义务教育均衡发展通过国家督导评估。四川职业护理学院一期完工，四川工业科技学院升格为本科学院，天一学院和指南针职业技术学校首批入驻师生1万人。公立医院改革稳步推进，分级诊疗全面实施，县级公立医院托管乡镇卫生院试点工作成效明显，全科医生团队签约服务全域推进，取消药品加成累计为群众让利8300多万元。广汉、罗江血吸虫病传播阻断达标。文化惠民工程扎实开展，建成331个省级“幸福美丽新村（社区）文化院坝”，举办德阳灯会、端午龙舟赛等民俗文化活动。体育事业不断发展，新建全民健身苑15个、农民健身工程113个，在“省十二运会”上取得佳绩。全市人口自然增长率1.48‰，单独两孩办证5289例。

社会治理迈出新步伐。“平安中国示范区”创建深入开展，社会治安防控网络不断完善，刑事案件下降8.9%。“法治德阳”扎实推进，“法律七进”及村规民约“中江经验”在全省推广。完成第九届村（居）民委员会换届选举，全面推行村民议事会议制度。在全国首创“邻里乡亲互助会”，政府救济与民间互助得到有机结合。在居家养老、社区服务等领域探索政府购买社会组织服务。加强群众和信访工作，“12345”为民服务平台投入运行。道路、生产、食品、药品等安全形势进一步好转，建成19个省级安全社区。防灾减灾和突发事件处置能力不断提升。国防动员、民兵预备役和“双拥”工作继续加强。

五、强化政府自身建设，行政效能不断提高

党的群众路线教育实践活动成效明显。以“为民、务实、清廉”为主题，聚焦“四风”问题，强化正风肃纪，开展“9+2”专项整治，狠抓问题整改落实，政府机关作风持续好转，服务效率明显提升，一批联系服务群众“最后一公里”问题得到切实解决。严控“三公”经费，全市同比减少4663万元、较预算下降18.5%。加强文山会海治理，市政府文件和全市性会议分别减少14.7%、7.8%。

依法行政深入推进。自觉接受市人大及其常委会的法律监督、工作监督和市政协的民主监督，办理人大代表建议109件、政协提案210件。全面推进法治政府建设，坚持政府常务会学法，普遍建立法律顾问制度，调整充实市政府法律顾问团，村（社区）全部配备法律顾问，4个村被授予“全国民主法治示范村”，我市行政复议工作经验在全国交流。

政务服务得到提升。深化“两集中两到位”，清理审批前置条件1254个，横向并联审批和纵向联动审批机制进一步完善。建成“一目录、一厅、两平台”，行政权力依法规范公开运行平台开通运行，清理并晾晒“权力清单”6403项。全程代办投资项目375个，立项投资额1539亿元；建立投资项目统一收费、规范减免机制，办理减免金额1207万元。搭建公开规范的涉审中介平台，试点地区的政府投资项目中介服务费下浮50%、服务效率提升70%。

统计调查、档案、民族宗教、住房公积金、妇女儿童、青年、老龄、残疾人、人防、保密、地方志、气象、红十字会等工作取得新进展。

各位代表，过去的一年，我们在“稳增长”中增添了新动力，在“调结构”中迈出了新步伐，在“促改革”中实现了新突破，在“惠民生”中取得了新业绩。成绩来之不易，归功于全市人民的共同努力。在此，我代表市人民政府，向市人大代表、政协委员和离退休老同志，向各民主党派、工商联、无党派人士、人民团体和全市人民，向驻地人民解放军、武警官兵和公安干警，向所有关心、支持德阳发展的海内外朋友，表示衷心的感谢并致以崇高的敬意！

同时，我们也清醒看到，经济下行压力仍然存在，转型升级任务还很艰巨；工业发展质量和效益有待提高，服务业发展水平及层次需要提升，农业发展方式亟需转变；投资增长后劲不足，项目支撑作用还需增强；中心城市聚集辐射力不足，区域经济竞争力有待提升；财政持续增收困难，收支平衡压力突出；社会稳定压力依然较重，社会治理仍需进一步加强；政府职能转变还没有完全到位，发展环境还需优化。对此，我们将在今后工作中加倍努力，积极解决。

2015年工作安排

今年是全面深化改革的关键之年，也是全面完成“十二五”规划的收官之年。当前，我市经济社会发展面临不少困难和挑战，经济下行压力较大、结构调整阵痛显现、风险和矛盾化解艰难。同时，我们也要看到，德阳正处于工业化、城镇化“双加速”时期，发展潜力大，发展基础好，发展动力足。我们要坚定信心，积极作为，以改革开放的新思路、转型升级的新举措，抢抓新常态发展中的新机遇，赢得经济社会发展的新成果。

今年政府工作的总体要求是：全面贯彻党的十八大、十八届三中四中全会和省委十届四次五次全会精神，特别是习近平总书记系列重要讲话精神，按照中央和省委经济工作会议要求，主动适应经济发展新常态，坚持稳中求进工作总基调，深入实施“三大发展战略”和“一三五八”发展战略，突出产业带动、内需拉动、创新驱动、改革推动，加强民生保障，强化风险防控，促进全市经济持续健康发展、社会和谐稳定。

今年经济社会发展主要预期目标是：地区生产总值增长8%左右；规模以上工业增加值增长9%左右；全社会固定资产投资950亿元左右；一般公共财政预算收入85.67亿元；社会消费品零售总额增长12%左右；城镇居民人均可支配收入增长9%左右；农民人均纯收入增长10%左右；城镇登记失业率控制在4%以内；节能减排达到国家和省的要求。

一、坚持稳中求进，提高经济发展质量效益

加大产业结构调整力度，突出增量优质、存量优化，大力发现培育新增长点，在更高层次上加快产业转型升级。

狠抓工业提质增效。坚定不移实施工业强市战略。推动传统产业向中高端迈进，引导机械行业向研发设计、智能制造、营销和服务等高附加值环节延伸；引导化工行业向精细化工、特种化工、清洁生产方向发展；引导食品产业紧盯市场调整结构。加快发展战略性新兴产业，重点培育航空与燃机、新能源汽车、新材料三大高端成长型产业，大力推进电科特飞、东汽燃机、明日宇航、什邡烟厂技改等20个重大项目建设。推动园区调位升级，加快德阳广汉国家高新区创建，支持中江创建省级高新区，抓好通航产业园、石墨烯产业园、石油天然气装备产业园建设。促进工业化信息化融合，加快智慧城市建设，推进“工业云制造”基地、“工业云服务”平台和“云计算中心”建设。加大企业服务力度，支持企业拓展市场、技术创新、管理创新和经营模式创新，“一企一策”帮助困难企业。

提升发展现代服务业。把发展现代服务业作为调整产业结构的主攻方向，研究出台更具突破性的政策措施，促进服务业比重提高、业态提升。扎实推进省级现代服务业综合改革试点，突出抓好“五大新兴先导型服务业”。完善现代物流体系，支持大型制造企业剥离和外包物流业务，发展壮大物流龙头企业。大力发展电子商务，加快当当网西南电子商务产业园、普洛斯电子商务物流产业园建设。打造城市商圈、特色商业街，抓好服务业重大项目建设。积极引进大型会展专业公司，促进会展经济发展。引导房地产市场稳定健康发展。提升旅游设施，打造文化游、乡村游、古镇游、赏花品果游，彰显旅游特色。

加快推进农业现代化。大力发展农业产业化，积极推进多种形式适度规模经营，发展各类新型农业经营主体。稳定发展粮食生产，开展整县整乡粮油高产创建，继续抓好现代农业、林业和畜牧业重点县建设，实施现代农业千亿示范工程和粮经复合种植基地建设工程。调优农业结构，培育壮大粮油、蔬菜、生猪等主导产业基地，巩固发展小家禽、水果、干果、中药材等区域性特色产业，构建优势区域布局和专业生产格局。加强耕地质量建设。加大农业资金投入，推进农业多功能开发，拓宽农民增收渠道。

二、坚持改革创新，催生发展新动力新活力

把改革创新贯穿于经济社会发展各领域各环节，全面落实深化改革“规定动作”，大胆探索德阳改革“自选动作”，不断释放改革红利。

推进重点领域改革。认真落实已出台的改革措施，抓紧制定具体改革方案，有序推进各项改革。完善现代市场体系，扎实推进信用体系建设，深化商事登记制度改革，坚持宽进严管，逐步推行先照后证。深化以管资本为主的国资监管体系改革，推进市属国有停产企业“人资分离”改革，大力发展以现代产权制度为基础的混合所有制经济，加快国有企业生活区移交属地社区管理工作。深化行政管理体制改革，全面推行“四项清单”，加大行政审批权限精简下放力度。推动优化行政层级和行政区划设置改革。推进财税体制改革，深化财政专项资金管理改革，完善市、区分税制财政体制，合理控制政府性债务，清理规范税收等优惠政策。完成公务用车改革。深化农村改革，加快农村产权确权颁证，推进土地经营权流转，建立农村产权交易（流转）服务体系；创新农业投融资机制，探索和扩大农村土地流转收益保证贷款试点。

着力激发创新活力。大力实施创新驱动发展战略，扎实推进创新驱动发展体制改革，增强产业内生增长动力。提升企业自主创新能力，新增10家高新技术企业、20家创新型企业。围绕产业链部署创新链、配套资金链，组织实施80项创新驱动发展专项项目。促进科技成果转化，推动企业与川大产业技术研究院等科研单位开展项目对接，抓好中科育成中心等创新载体建设。加快推进德阳市科技馆建设。推动科技金融结合，开展股权融资、知识产权质押融资、债券融资。深入推进“双百工程”，引进培育一批高层次创新创业人才特别是领军人才。继续开展“国家知识产权示范城市”创建，抓好“国家创新驱动助力示范区”工作。

深入推进开放合作。以开放促改革、以开放促创新。把握“一带一路”和长江经济带机遇，加快德阳综合保税区筹建，提升海关、出入境检验检疫服务能力 ，加强与沿海沿江沿边城市通关协作，培育内陆开放新优势。深度融入成都经济区，大力推进成德同城化，加快成德工业园、德阿产业园建设，支持罗江与绵阳开展产业合作。突出产业链关键环节和缺失环节招商，积极参加西博会、中外知名企业四川行等活动，确保到位内资650亿元，利用外资2.1亿美元。服务企业“走出去”，提升发展出口贸易和服务贸易，扩大机电产品、钛白粉等优势产品出口，完成进出口总额36亿美元左右。抓好对口援建芦山县、阿坝县和若尔盖县工作，加强与北京、江苏援建省市的联系，深化与珠三角、长三角等区域合作。

大力发展县域经济和民营经济。加强分类指导，支持各县（市、区）突出特色、错位发展，充分释放县域经济活力。坚持统筹协调，加大全市在规划、招商引资、项目建设、环境保护、扶贫攻坚等方面的协调力度。毫不动摇支持民营经济发展，严格落实财政扶持、税收优惠、用地保障等政策措施，促进大众创业、万众创新。扶持中小微民营企业发展壮大，鼓励民营企业参与国有企业改制重组，激发民资民智民力的活力和创造力。积极帮助民营企业破解发展中的难题。

三、强化投资促进，不断释放投资消费潜力

发挥好投资的关键作用和消费的基础作用，促进政府投资与民间投资共同发力。

切实加快项目建设。强力推进169个省市重点项目，确保完成年度投资265.5亿元。抓好绵茂公路建设，配合做好成南铁路建设，实现成德大道、中金快速通道通车，开工成都经济区外环线高速公路东段、加速北段招标，开展德中快速通道前期工作，推进农村公路建设。加快华强沟、凯江湖、八角等水库建设，开工石泉水库，做好三渔、丰家桥、姜家桥等水库前期工作。狠抓项目开工竣工，形成更大的投资额和实物工作量。

完善投融资服务体系。主动对接国家政策支持和投资结构调整方向，力争更多项目纳入国省“盘子”。发挥政府资金引领作用，用好基本建设资金和投融资平台，引导更多社会资本进入公用事业、民生保障、基础设施、节能环保等领域。加强金融服务创新，搭建银政企对接平台，引导金融机构加大对科技型企业、中小微企业的支持力度，鼓励金融机构到乡镇增设服务网点。加快推进农村信用合作社改制为农村商业银行，支持德阳银行改革发展。大力发展现代保险服务业，探索保险资金支持重大项目融资方式。开展多层次资本市场直接融资，推动符合条件的企业发行公司债、中小企业集合债券。依法严厉打击非法集资，营造良好金融生态环境。

着力激发消费潜力。借成德绵乐城际铁路投运之机，大力发展特色餐饮、休闲旅游等产业，吸引外来消费。积极参与“惠民购物全川行”、“川货全国行”、“万企出国门”三大活动，拓展市场需求。大力发展信息消费、健康养老、旅游文化、汽车消费等拉动力强的消费热点。创新发展网络购物、信贷消费、租赁消费等新型消费业态。搭建各类促销平台，办好“德阳造”名优特新产品展销会等活动，繁荣节假日市场。

四、注重统筹推进，加快新型城镇化发展

新型城镇化是最大的结构调整，要坚持走以人为核心的新型城镇化道路，力争城镇化率提高1.6个百分点。

提升中心城区品质。打造中心突出、功能完善、生态优美的区域中心大城市，增强聚集力、辐射力、带动力。科学推进城市新区建设，完善新区规划体系，强化产城互动，加速产业、人口聚集，重点抓好漓江路大桥、长江西路延长线、漓江路和沱江路下穿宝成线一期等重大基础设施建设。加大旧城和棚户区改造力度，注重保护和传承历史文化，加强立体交通和地下管网体系建设，发展城市公交、城乡公交、城际公交。构建城市管理长效机制，巩固创国卫成果，坚决打击违法建设行为。加强城市绿地系统建设，增加“邮票绿地”和“袖珍广场”，将旌东新区起步区建设成为国家标准的绿色生态城区。

推动城镇群协调发展。加快全市域一体化进程，推进“1+5+X”城镇群建设，构建“三环五廊”市域空间结构，搞好湿地资源保护、绿道网建设。加快县域城市发展，完善县域快速通道和城际间一体化交通联系，联动抓好产业园区和县域城市新区建设，提高县域城市综合承载力。抓好重点镇和特色镇建设，结合资源禀赋明确发展定位，强化产业支撑，探索整镇推进新型城镇化，抓好“百镇建设试点镇”工作。

加快建设幸福美丽新村。坚持规划先行，集中连片推进新农村建设，新（改）建幸福美丽新村50个、新村聚居点160个。坚持产村相融，因地制宜发展特色产业。加大新村建设投入，整合各类项目资金，完善新村建设资金分担机制，抓好农村饮水安全、电网改造升级、沼气建设等工程，改造农村危房1208户，帮助4.5万农村贫困人口改善生产生活条件。积极解决农村空心化、老龄化、“三留守”等问题。提高农民素质，推动“物的新农村”和“人的新农村”建设齐头并进。

推进农业转移人口市民化。探索建立农业转移人口市民化成本分担机制，积极引导符合条件的农民到城镇落户。推进城镇基本公共服务常住人口全覆盖，继续实施“农民工住房保障行动”，完善并落实好农民工参加各项社会保险政策，保障农民工随迁子女平等接受教育，加强农民就业增收技能培训。

五、突出生态文明，打造宜居生态绿色家园

生态环境是生存之本、发展之基和民生之需。实施最严格的源头保护、总量控制、责任追究制度，提升环境质量。

强化节能减排。抓好节能减排财政政策综合示范城市工作，推动典型示范项目实施。将节能减排与产业结构调整相结合，鼓励工业项目采用节能减排先进技术工艺，淘汰落后产能企业20余户。切实抓好主要污染物总量减排，扎实推进主要污染物总量指标有偿使用，建立排污权交易市场，全面开展排污许可证核发工作。

高效利用资源。建立项目用地退出机制，严格执行用地投资强度标准，用好增量、盘活存量，提高土地资源利用效益。突出循环经济导向，抓好国家资源综合利用“双百工程”，提高磷石膏、秸秆等资源的综合利用率，探索综合利用产业化路径。建成天元城市生活污水处理厂，完善污水处理厂配套管网。加强垃圾源头治理和控制，统筹规划建设城市生活垃圾焚烧发电厂，推进生活垃圾资源化、无害化处理。

加强生态环保。狠抓大气污染整治，抓好工业污染脱硫脱硝、建筑扬尘、餐饮油烟、燃煤锅炉淘汰、油气回收等大气污染防治工作，强化机动车尾气治理，加强秸秆禁烧联防联控。深入推进水环境治理，重点抓好沱江流域德阳段水污染综合防治，加强饮用水水源地保护。继续实施全流域河道禁养，治理农村面源污染。巩固天然林保护、退耕还林、森林城市创建等成果，抓好林业有害生物普查，建立健全生态补偿机制，搞好重大地质灾害隐患治理和水土保持工作。

六、强化民生改善，切实保障和增进民生福祉

突出富民、惠民、利民导向，深入实施“十大民生工程”和新增民生实事，让发展成果更好更公平惠及群众。

全面落实惠民政策。推进就业创业体制机制改革，积极创建省级创业型城市，重点解决好高校毕业生、困难群众和进城农民的就业问题，新增就业3.2万人。推进社会保障制度改革，全面启动全民参保登记试点，加快城乡医保整合，开展城镇职工大病保险工作，逐步实现城乡居民医保政策、待遇、系统结算“三统一”。健全最低生活保障标准合理增长机制。推进收入分配制度改革，加大工资集体协商力度，推进机关事业单位养老保险制度和工资制度改革。继续实施“百万安居工程”，开工保障性安居工程29209套，其中棚户区改造27757套。抓好社会救助、防灾减灾、社会福利、公益慈善体系建设。

加快发展社会事业。实施第二轮学前教育三年行动计划，加大幼儿园建设力度。推进义务教育均衡发展，确保旌阳、什邡、中江、罗江通过县域义务教育基本均衡发展督导评估，新（改）建小学38所。构建产教融合的现代职教体系，全力推进四川护理职业学院、西南财大天府学院建设。强化县级公立医院改革，深化取消药品加成工作，完善分级诊疗制度，鼓励城市医疗机构与县级医院和乡镇卫生院进行技术合作，试行医师多点执业。搞好重大疾病和地方病防控，确保旌阳、中江血吸虫病传播阻断达标，强化艾滋病教育、预防和控制。稳妥推进生育新政，促进人口长期均衡发展。广泛开展群众性文化创建和全民阅读活动，实施重点文化惠民工程，大力发展文化产业。开展全民健身活动，打造户外休闲运动基地，提高竞技体育水平。

创新和加强社会治理。全面推进社会治理体制机制建设，实现依法治理、源头治理、综合治理。改进社会治理方式，建立完善村（社区）民情恳谈、民主议事和民主监督制度。支持社会组织发展，简化社会组织登记手续，加快实施政社分开，扩大政府购买社会组织服务。深入开展法治宣传教育，推进覆盖城乡居民的公共法律服务体系建设。深入推进“平安中国示范区”建设，创新预防和化解社会矛盾体制，强化社会治安防控体系和应急能力建设，建立食品源产地可追溯和质量标识制度，坚决遏制重特大安全事故，抓好15个省级安全社区建设。做好国防动员、民兵预备役和“双拥”工作。

七、着力转变职能，建设法治政府和服务型政府

把严格依法办事贯穿于政府工作的各个环节，大力提升治理能力和服务能力，努力打造人民满意政府。

推进依法行政。坚持在党的领导下、在法治轨道上开展工作，加快建设职能科学、权责法定、执法严明、公开公正、廉洁高效、守法诚信的法治政府。自觉接受市人大及其常委会的法律监督和工作监督、市政协民主监督、社会舆论监督。强化政府内部层级监督，加强行政监察、财政监督和审计监督。健全依法决策机制，把公众参与、专家论证、风险评估、合法性审查、集体讨论决定作为重大决策必经程序。强化规范性文件备案审查。推进综合执法和行政裁量权基准制度，细化、量化行政裁量标准。

提高行政效能。强化政府执行职责，健全行政责任体系，改进考核评价办法，优化绩效管理体系，着力提升执行力。深化“为民服务阳光行动”，发挥行政权力依法规范公开运行平台作用，促进权力依法、公开、便民、高效运行。健全并联审批、网上审批、“一窗进出”运行机制；推进政务服务标准化；完善四级政务服务体系功能。健全投资项目全程代办机制。规范中介服务平台，建立信用评价和清退机制。

强化勤政廉政。巩固党的群众路线教育实践活动成果，持之以恒改进作风，坚决纠正不作为、乱作为，坚决克服懒政、怠政，坚决惩处失职、渎职，坚决防止“四风”问题反弹。深入贯彻省委“两个意见”，严格落实党风廉政建设责任制，加强工程建设、公共资源交易等重点领域监督。严格落实《党政机关厉行节约反对浪费条例》和省、市各项禁令，坚持勤俭办事，进一步压缩“三公”经费。

各位代表，新常态意味着新考验，新常态蕴藏着新机遇。让我们紧密团结在以习近平同志为核心的党中央周围，在省委、省政府和市委的坚强领导下，创新求进，拼搏实干，为实现全市“两个跨越、两个率先”的目标而努力奋斗！
